# A Cost-Effective Relative Humidity Sensor Based on Side Coupling Induction Technology

**DOI:** 10.3390/s17050944

**Published:** 2017-04-25

**Authors:** Yingzi Zhang, Yulong Hou, Wenyi Liu, Huixin Zhang, Yanjun Zhang, Zhidong Zhang, Jing Guo, Jia Liu, Liang Zhang, Qiu-lin Tan

**Affiliations:** 1 Key Laboratory of Instrumentation Science & Dynamic Measurement, Ministry of Education, North University of China, Taiyuan 030051, China; zhangyingzinuc@163.com (Y.Z.); liuwenyi@nuc.edu.cn (W.L); zhanghx@nuc.edu.cn (H.Z.); zhangyanjun@nuc.edu.cn (Y.Z.); zdzhang@nuc.edu.cn (Z.Z.); 20150156@nuc.edu.cn (J.G.); 18724920710@163.com (J.L.); zhangliang_ty@163.com (L.Z.); tanqiulin.99@163.com (Q.T.); 2 Science and Technology on Electronic Test & Measurement Laboratory, North University of China, Taiyuan 030051, China

**Keywords:** relative humidity sensor, side coupling induction technology, agarose, twisted macro-bend coupling structure

## Abstract

A intensity-modulated optical fiber relative humidity (RH) sensor based on the side coupling induction technology (SCIT) is presented and experimentally demonstrated. The agarose gel and the twisted macro-bend coupling structure are first combined for RH sensing applications. The refractive index (RI) of the agarose gel increases with the increase of the RH and is in linear proportion from 20 to 80%RH. The side coupling power, which changes directly with the RI of the agarose gel, can strip the source noise from the sensor signal and improve the signal to noise ratio substantially. The experiment results show that the sensitivity of the proposed sensor increases while the bend radius decreases. When the bend radius is 8 mm, the sensor has a linear response from 40% to 80% RH with the sensitivity of 4.23 nW/% and the limit of detection of 0.70%. A higher sensitivity of 12.49 nW/% is achieved when RH raises from 80% to 90% and the limit of detection decreases to 0.55%. Furthermore, the proposed sensor is a low-cost solution, offering advantages of good reversibility, fast response time, and compensable temperature dependence.

## 1. Introduction

Nowadays, relative humidity (RH) sensing is of great significance in a wide range of applications, including warehousing goods maintenance, cultural relic preservation, manufacturing process control, agricultural management and many other fields [1,2]. Accordingly, various technological approaches have been proposed to achieve RH measurement [3,4,5,6]. Optical fiber humidity sensors, compared with their electronic counterparts, exhibit certain advantages of fast response time, immunity to electromagnetic interference, and remote operation [7,8].

Currently, most optical fiber RH sensors in the reported literature are achieved by the wavelength-modulated technique or intensity-modulated technique [9]. The wavelength-modulated RH sensors aim to measure the wavelength changes of the absorption peak which vary according to the ambient humidity. To generate absorption peaks, a variety of structures—including Fabry-Perot resonator [10], in-fiber Mach-Zehnder interferometer [11], fiber Bragg gratings (FBGs) [12,13,14], and so on—have been applied. The wavelength-modulated technique always involves complicated processing technology or equipment, such as high-resolution optical spectrum analyzers, which increase the cost of fabrication and the complexity of operation. Consequently, the intensity-modulated technique shows a good potential to achieve a RH sensor with high performance and low cost [15,16,17]. As the intensity-modulated sensors are easily affected by the external environment, various methods are designed to diminish or eliminate the interferences. For example, a reference POF is adopted in a refractive index sensor to remove the source power variation [18]. The dark field characteristics of a twisted macro-bend coupling structure (TMBCS) are used to improve the signal to noise ratio, which realizes the liquid level detection in [19] and displacement sensing in [20].

In optical fiber RH sensors, hygroscopic materials are often selected to coat on the surfaces or end faces of the fiber and modulate the propagating light. The characteristics of RH sensors can make a big difference depending on the different choices of coating materials. For example, the tapered plastic optical fiber (POF) coated with agarose, hydroxyethylcellulose/polyvinylidenefluoride (HEC/PVDF), and seeded zinc oxide (ZnO) nanostructures show different humidity sensing performances and agarose is demonstrated to be a suitable selection with good sensitivity and linearity between 50% and 80% [21].

In this paper, a cost-effective RH sensor based on the SCIT is proposed. Here we first combine the agarose gel with the TMBCS to design a new RH sensing structure and the RH measurement is achieved by the tunable coupling power within the TMBCS. The fabrication process of the sensor is inexpensive and simple. The influences of the coating material and bend radius on the proposed sensor are experimentally investigated. The sensor turns out to be a good design with high sensitivity, good reversibility, fast response time, and remediable temperature dependence.

## 2. Working Principle

**Figure 1:**
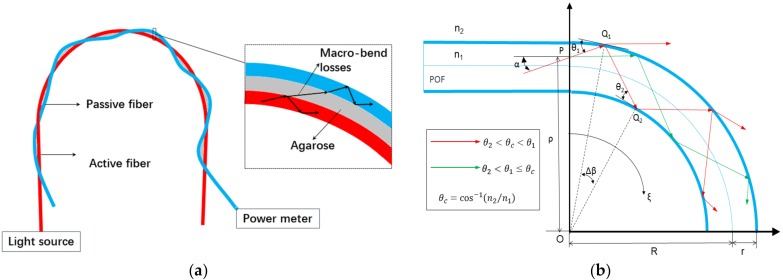
(**a**) Schematic diagram of the proposed RH sensor; (**b**) Geometrical model of the macro-bend active fiber.

The schematic diagram of the proposed RH sensor is shown in Figure 1a, which consists of two plastic optical fibers (POFs) and the agarose gel. Two untreated naked POFs are tightly twisted and then bent to a semicircle, forming a TMBCS. The fiber connected to the light source is called the active fiber and another fiber connected the power meter is called the passive fiber. The agarose gel is filled into the gap between the active fiber and the passive fiber, whose porosity allows it to absorb water molecules and change its refractive index (RI).

The principle of the proposed sensor can be described as the side coupling induction technology (SCIT), which means the humidity sensing could be achieved by measuring the change of side coupling power modulated by external humidity. One advantage of the SCIT is based on the fact that the macro-bend losses from the active power coupled to the passive fiber will not be affected by the fluctuation of light source since the light source energy is mainly concentrated in the core and the fluctuation of the coupled portion is negligible [14]. Another advantage is the tunable property of the side coupling power generated from the agarose gel whose RI shows a linear change with respect to RH from 20 to 80% RH [22].

As shown in Figure 1b, the macro-bend section of the fiber is displayed where the ray tracing method has been employed to analyze the light losses [23]. According to the Fresnel formula, the reflection takes place on the interface of medium with different RI. For POFs (Mitsubishi, SK40) we select here, as the thickness of the cladding (10 μm) is considerably small by contrast to the diameter of the core (980 μm), the core-cladding interface and the cladding-agarose interface have been simplified to a core-agarose interface here.

In Figure 1b, light propagates in the straight section of the fiber via total internal reflection, and the propagating angle α is required to be smaller than $\theta_{c}$. $\theta_{c}$ is the critical angle and can be represented as

(1) $$\theta_{c}=\cos^{-1}\left(\frac{n_{2}}{n_{1}}\right)$$

where $n_{1}$ and $n_{2}$ are the RI of the core and the agarose gel. When light propagates in the macro-bend section, a portion of light are refracted at some points of the core-agarose interface where the propagating angle exceeds the critical angle $\theta_{c}$, which results in the macro-bend losses. As can been seen in Figure 1, an arbitrary incident ray entering the bent section with a propagating angle α and a location ρ. In $\Delta {\mathrm{OPQ}}_{1}$ and $\Delta OQ_{1}Q_{2}$, the geometric relationships can be deduced by

(2) $$\rho \cos\alpha =\left(R+r\right)\cos\theta_{1}=\left(R-r\right)\cos\theta_{2}$$

(3) $$\theta_{1}=\cos^{-1}\left(\frac{\rho \cos\alpha }{R+r}\right)$$

(4) $$\theta_{2}=\cos^{-1}\left(\frac{\rho \cos\alpha }{R-r}\right)$$

where *R* is the radius of the macro-bend section of the fiber and *r* is the radius of the fiber. The transmission coefficient, which can be used to estimate macro-bend losses of each ray, can be calculated from the equation [18]

(5) $$T=\{\begin{array}{cc} \frac{4\sin\theta_{1}}{\sin\theta_{c}}{\left[\frac{\sin^{2}\theta_{1}}{\sin^{2}\theta_{c}}-1\right]}^{1/2}, & \theta_{2}<\theta_{c}<\theta_{1}\\ 0, & \theta_{2}<\theta_{1}\leq \theta_{c} \end{array}$$

where all rays for which $\theta_{1}\leq \theta_{c}$ are assumed to be totally reflected and no power is lost at these points. According to the Lambert–Beer law, the output power $P_{o1}$ of the active fiber can be calculated from

(6) $$P_{o1}=P_{i}exp\left(-\gamma \xi \right)$$

(7) $$\gamma =\frac{T}{\Delta \beta }=\frac{T}{\theta_{1}-\theta_{2}}$$

where $P_{i}$ is the input power, γ is the attenuation coefficient for a unit length of the fiber, and ξ is the angular displacement of the curvature of the macro-bend section. Hence, the transmission power $P_{t}$ of the light is given by

(8) $$P_{t}=P_{i}-P_{o1},$$

and the transmission ratio of the light can be represented as

(9) $$\eta =\frac{P_{t}}{P_{i}}=\frac{\int_{R-r}^{R+r}\int_{-\theta_{c}}^{\theta_{c}}\left[1-exp\left(-\gamma \xi \right)\right]d\rho d\alpha }{\int_{R-r}^{R+r}d\rho \int_{-\theta_{c}}^{\theta_{c}}d\alpha }.$$

An energy coupling region is established between the pair of twisted POFs and a portion of the transmission power will be coupled to the passive fiber. We assume the side coupling power will not be lost in the straight section and the output power $P_{o2}$ of the passive fiber can be expressed as

(10) $$P_{o2}=P_{i}\eta C,$$

where C is the coupling coefficient.

From Equation (9), it can be deduced that the transmission ratio η is modulated by R and $n_{2}$. When the macro-bend radius R decrease or the RI of the agarose gel $n_{2}$ increase, η will increase, resulting in more macro-bend losses. According to the equations above, if the macro-bend radius is a fixed value, the output power of the passive fiber will change directly with the RI of the agarose gel which increases with the increase of the RH. This principle is called the SCIT and the RH sensor we proposed bases on it.

## 3. Sensor Fabrication and Experimental Setup

### 3.1. Probe Fabrication

First, two untreated naked POFs (Mitsubishi, Tokyo, Japan, SK40) were twisted and bent to a loop with a selected radius. Since the concentration of the agarose solution influences the sensitivity of the agarose coated probe, 0.5 wt % agarose gel with higher sensitivity is selected in contrast to 1 wt % and 1.5 wt % [16]. A magnetic stirrer including a heater (MS-H280-Pro, Dragonlab, Beijing, China) was applied to heat up the distilled water in a beaker to 65 °C and dissolve 0.5 wt % Agarose (A6013, Sigma Aldrich, St. Louis, MO, USA) in it. Then the hot solution was injected into the gap between the two POFs with a syringe. At last, all coated probes were left to dry for 24 h at room temperature.

### 3.2. Experimental Setup for Measurement

**Figure 2:**
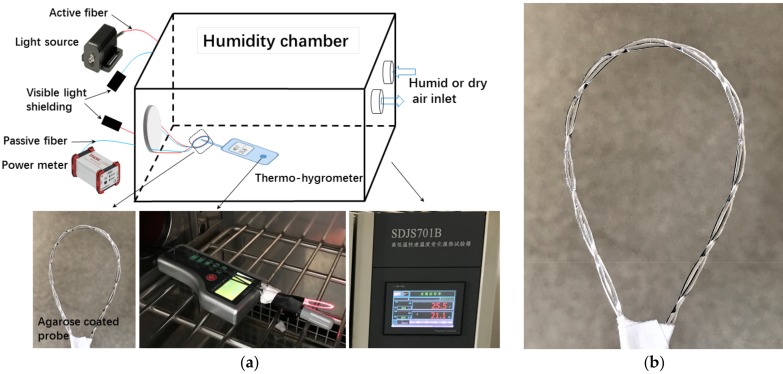
(**a**) Experimental setup of the proposed RH sensor; (**b**) The photo of the agarose coated probe.

The experimental setup of the RH sensor and the photo of the agarose coated probe are shown in Figure 2. The setup consists of a 660 nm fiber-coupled LED (M660F1, Thorlabs, Newton, NJ, USA) light source, an optical power meter (PM100USB, Thorlabs), a humidity chamber (SDJS701B, Chongqing SD Equipment, Chongqing, China), a thermos-hygrometer (AH8008, AOSONG, Guangzhou, China), and an agarose coated probe. The probe was fixed inside the chamber and the thermos-hygrometer was used to give a reference. The temperature set inside the chamber is 25 °C. The light source power was set at 35 mW. Through the constant value control of the chamber, the humidity response of the agarose coated probe was studied by adjust the humidity from 40% to 90% and then back to 40% gradually. The time step of the humidity settings was the default value. The sampling period of the power meter and thermos-hygrometer was 1 s. To avoid the interference of visible lights, the POFs except for the probe were put into the black jacket and so were the free ends.

## 4. Results and Discussion

### 4.1. With or Without Agarose

**Figure 3:**
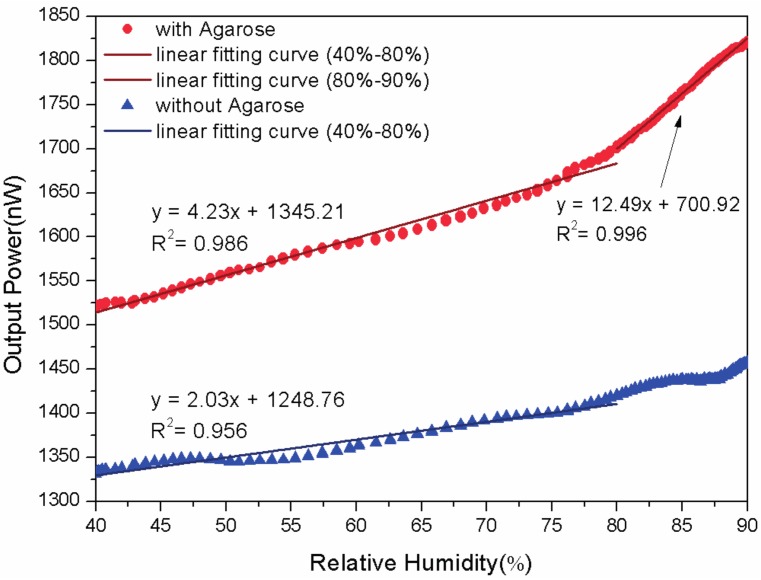
The humidity response of the proposed sensor with and without the agarose gel.

The humidity response of the sensor with and without the agarose gel is observed and the result is depicted in Figure 3. The bend radius of these probes here is 8 mm. When RH raises from 40% to 80%, the probe without agarose has a sensitivity of 1.49 nW/% with a slope linearity of 80.00%. The limit of detection is 2.92% and it is calculated by dividing the standard deviation with the sensitivity. Correspondingly, the probe with agarose has a higher sensitivity of 4.23 nW/% with a better linearity of 99.30%. The limit of detection decreases to 0.70%, which means the agarose coated probe is more efficient. Reason for that is the higher RH means the higher RI of agarose in the gap between the two fibers, which leads to an apparent increase of the side coupling power. The performance comparison is summarized in Table 1. When RH raises from 80% to 90%, the sensitivity of the agarose coated probe increases to 12.49 nW/% with the linearity of 99.8% and the limit of detection decreases to 0.55%. It can be observed that the proposed sensor has a maximum sensitivity of between 80% and 90% and this quality could be adopted for potential applications in high-humidity atmosphere.

### 4.2. Different Radiuses

**Figure 4:**
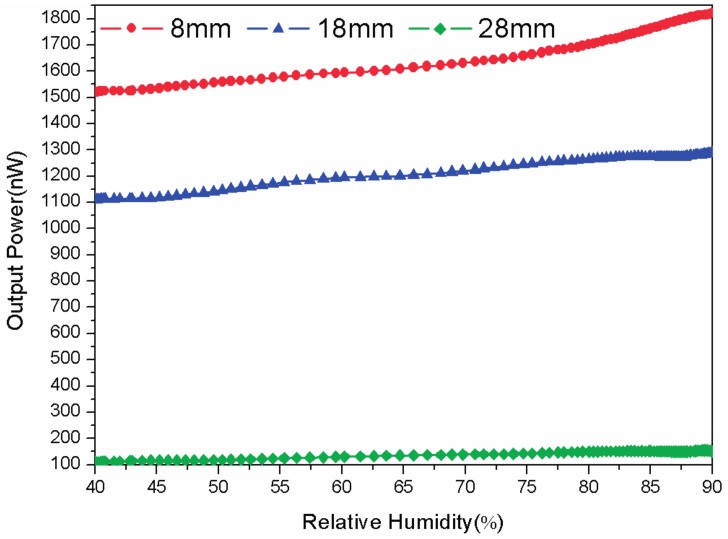
The humidity response of the proposed sensor with different radiuses.

In Figure 4, the performances of the sensor with three different curvature radiuses (8, 18, and 28 mm) are investigated. As expected when the radius decreases the coupling power of the probe increases and so does the sensitivity. Therefore, to achieve a good performance of the sensor, we could choose a smaller bend radius within the capacity of the POFs and a curvature radius of 8 mm is selected here for further study.

### 4.3. Reversibility

**Figure 5:**
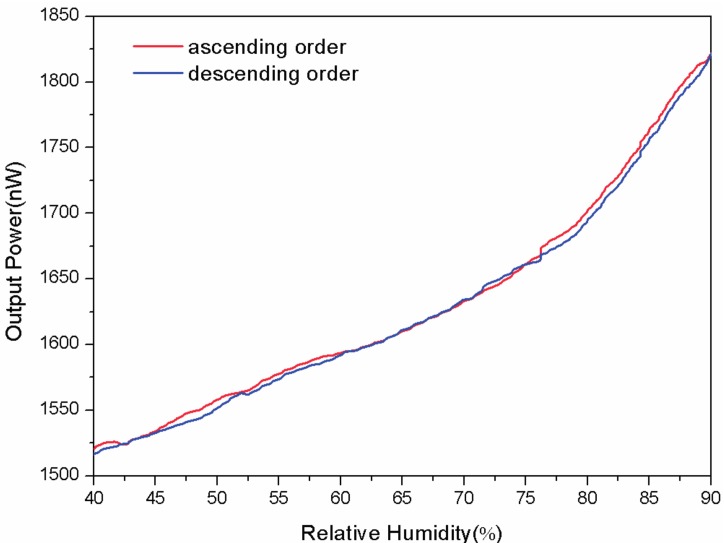
The reversibility of the proposed sensor with a bend radius of 8 mm.

The reversibility is an important factor to consider when evaluating a sensor. In Figure 5, the output power of the passive fiber is observed in the humidification-dehumidification cycle. As can be seen, the maximum difference is 8.1 nW which is small and acceptable compared with the full-scale output of 305.06 nW.

### 4.4. Response Time

**Figure 6:**
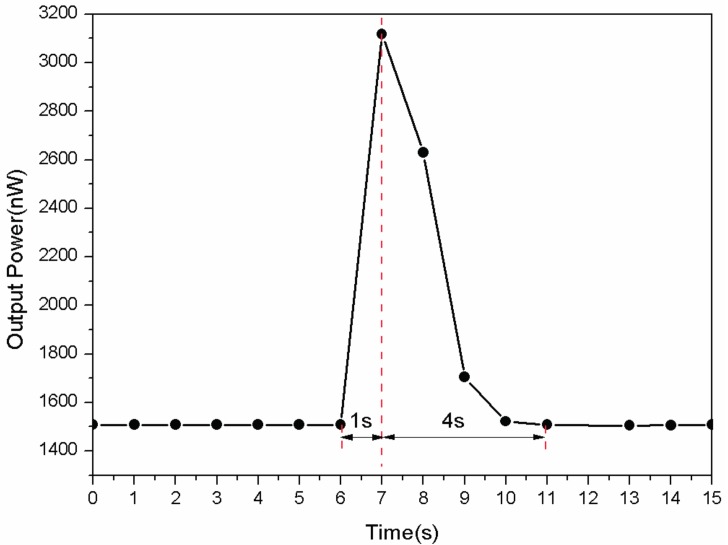
The response and recovery time of the proposed sensor with a bend radius of 8 mm.

As the adjustment speed of the humidity chamber is too slow to study the response time of the sensor, a step change of RH is achieved by exhale a 1 s long breath directly to the probe [9,14,15]. To evaluate the response time, this method could not give an accurate value but provide a reference. The ambient humidity during the experiment is 40%RH and the temperature is 25 °C. The result is shown in Figure 6. Limited by the detecting precision of the power meter, the response time is less than 1 s and the recovery time 4 s, which is much faster than traditional humidity sensors.

### 4.5. Temperature Dependence

**Figure 7:**
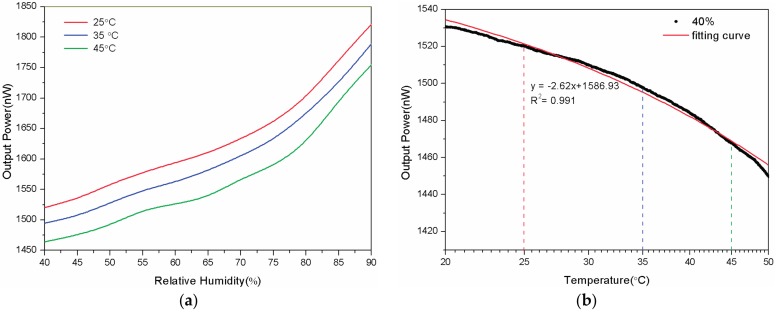
(**a**) The humidity response of the proposed sensor at 25, 35, and 45 °C, respectively; (**b**) The temperature dependence of the sensor at an ambient humidity of 40%RH.

Finally, the temperature dependence of the sensor is investigated. Since the thermo-optic (TO) coefficient of POFs is relative high compared to the one of GOFs and the agarose gel are temperature dependent, the result is a combined influence of both dependencies. As shown in Figure 7a, the beginning output power of the sensor is different while the temperature of the chamber is set at 25, 35, and 45 °C, respectively. However, the slope of the curves practically does not change, which means the sensitivity of the sensor is temperature-independent. The Figure 7b illustrates the change of the output power with the temperature gradually raising from 20 °C to 50 °C when the RH is set to 40%. The sensor exhibits a linear change with temperature varying from 20 °C to 50 °C. Therefore, a temperature compensation is easy to obtain like introducing a thermometer and so on.

Table 2 presents the performances of the proposed sensor compared with the previous RH sensor based on intensity-modulated technique. As mentioned previously, the proposed sensor shows a linear, reversible, and fast response with the humidity variation since the RI of the agarose gel change linearly and reversibly with respect to the RH [24]. The sensor also offers a wide humidity operating range because the agarose gel is humidity-sensitive in a wide RH range. It can be deduced that the performances of the SCIT based sensor are influenced mainly by the hygroscopic material we select. Hence, our future research will focus on studying different hygroscopic materials within the TMBCS and find out the one with the best performances for RH monitoring.

## 5. Conclusions

In conclusion, a cost-effective RH sensor based on the SCIT has been theoretically and experimentally investigated. The sensor is constructed by filling the agarose gel between the two POFs in the TMBCS, whose fabrication is simple and inexpensive. When the bend radius is constant, the side coupling power changes directly with the RI of the agarose gel influenced by the external humidity. The proposed sensor shows a linear change in the output power while the RH ranging from 40% to 80% and the sensitivity is 4.23 nW/%. The highest sensitivity of 12.49 nW/% is achieved from 80% to 90% and the limit of detection is 0.55%. The experimental results of the sensor show good reversibility, fast response time, and compensable temperature dependence.

## Figures and Tables

**Table 1:** Performance of the proposed sensor.

Performances	With Agarose	Without Agarose
Sensitivity (nW/%)	4.23	1.49
Linearity (%)	99.30	80.00
Std deviation (nW)	2.98	4.35
Limit of detection (%)	0.70	2.92

**Table 2:** Comparison between the proposed sensor and the cited RH sensor.

Reference	Structure of Fiber	Coating Material	Sensitivity	Sensing Range (%)	Response Time (s)
This paper	Twisted macro-bend coupling structure	Agarose	4.23 nW/%	40–90	1
[13]	Singlemode polymer fiber Bragg	None	0.23 mV/%	10–90	4.5
[14]	Side polished fiber	WS_2_ film	0.1213 dB/%	35–85	1
[15]	Side polished fiber	Reduced graphene oxide	0.31 dB/%	70–95	5
[16]	Tapered POF	HEC/PVDF	0.023 mV/%	50–85	none
[20]	Tapered POF	ZnO	0.0258 mV/%	50–85	none
[20]	Tapered POF	Agarose	0.0228 mV/%	50–85	none
